# Individual Differences in Implicit and Explicit Spatial Processing of Fractions

**DOI:** 10.3389/fpsyg.2019.00596

**Published:** 2019-04-05

**Authors:** Elizabeth Y. Toomarian, Rui Meng, Edward M. Hubbard

**Affiliations:** Department of Educational Psychology, University of Wisconsin–Madison, Madison, WI, United States

**Keywords:** spatial-numerical associations, SNARC, number line estimation, fractions, individual differences

## Abstract

Recent studies have explored the foundations of mathematical skills by linking basic numerical processes to formal tests of mathematics achievement. Of particular interest is the relationship between spatial-numerical associations—specifically, the Spatial Numerical Association of Response Codes (SNARC) effect—and various measures of math ability. Thus far, studies investigating this relationship have yielded inconsistent results. Here, we investigate how individual implicit and explicit spatial representations of fractions relate to fraction knowledge and other formal measures of math achievement. Adult participants (*n* = 105) compared the magnitude of single digit, irreducible fractions to ½, a task that has previously produced a reliable SNARC effect. We observed a significant group-level SNARC effect based on overall fraction magnitude, with notable individual variability. While individual SNARC effects were correlated with performance on a fraction number-line estimation (NLE) task, only NLE significantly predicted scores on a fractions test and basic standardized math test, even after controlling for IQ, mean accuracy, and mean reaction time. This suggests that–for fractions–working with an explicit number line is a stronger predictor of math ability than implicit number line processing. Neither individual SNARC effects nor NLE performance were significant predictors of algebra scores; thus, the mental number line may not be as readily recruited during higher-order mathematical concepts, but rather may be a foundation for thinking about simpler problems involving rational magnitudes. These results not only characterize the variability in adults’ mental representations of fractions, but also detail the relative contributions of implicit (SNARC) and explicit (NLE) spatial representations of fractions to formal math skills.

## Introduction

Recent efforts to understand predictors of mathematical achievement have begun to focus on the contribution of spatial skills in addition to numerical abilities. This initiative has widespread educational implications, as spatial ability in early teenage years predicts the eventual likelihood of pursuing advanced study in STEM (Science, Technology, Engineering and Mathematics) topics and careers in a STEM field (Shea et al., 2001; Wai et al., 2009). The combined development of spatial and numeracy skills are unique predictors of later mathematical success and other academic outcomes, with strong cross-domain links evident from early childhood (for a review, see Mix and Cheng, 2012). For instance, spatial skills at age 5 have been shown to predict standardized math scores at age 7 (Gunderson et al., 2012; Gilligan et al., 2017), and a number of spatial skills (e.g., mental rotation, visuospatial working memory) predict math performance throughout childhood. One possible account for these relationships is the close behavioral, cognitive, and neural link between numbers and space (e.g., Hubbard et al., 2005; Toomarian and Hubbard, 2018a).

These findings highlight just a few of the many factors that contribute to early mathematical understanding. Multiple numerical abilities likely serve as precursors to greater mathematical ability, though some may contribute more or less than others, with many competencies being closely related. For instance, in one specific study, preschool children’s approximate number sense and cardinality knowledge of number words both predicted later math achievement, and cardinality was found to mediate the relationship between approximate number and math achievement (Chu et al., 2015). Further investigation of these factors is certainly needed, particularly as they relate to classes of numbers such as fractions, which are believed to be a critical part of a strong foundation for numerical understanding and uniquely predictive of later algebra-readiness (Booth and Newton, 2012).

In the current study, we specifically investigated the relationship between measures that link spatial and numerical processing of fractions by using several measures of implicit and explicit spatial-numerical associations (SNAs). We then aimed to determine the unique contribution of these factors to multiple measures of formal math achievement, such as tests of fractions arithmetic and algebra.

### Spatial-Numerical Associations and the Link to Mathematics

Spatial and numerical cognition have been studied in conjunction since at least the 19th century (Galton, 1880), with mounting evidence that both evolutionary and cultural factors contribute to the widely-evidenced link between the two (for a review, see Toomarian and Hubbard, 2018a). The link between numbers and space is supported from a number of theoretical perspectives. The mental number line (MNL) theory suggests that people have an internal representation of a number line, along which numerical magnitudes extend horizontally in the direction congruent with their primary written language (e.g., left-to-right for English readers) (Dehaene et al., 1993). This internal conceptualization links numbers and space along a linear continuum. There is also theoretical support from a developmental perspective; one of the central claims of the integrated theory of numerical development (Siegler et al., 2011) is that solid mathematical understanding requires knowing that all numbers have magnitudes that can be spatially oriented and placed on number lines. Despite the theoretical basis for a link between spatial skills and numerical cognition, it is unclear whether SNAs directly influence complex cognitive functions such as mathematical thinking.

In order to measure the implicit link between numbers and space, researchers typically employ one of several behavioral tasks, the most common being a parity or numerical judgment task with spatially-coded responses. In the magnitude judgment task, participants indicate whether a number is larger or smaller than a standard reference number by using either a left- or right-side response key, while in the parity task participants indicate whether the given number is even or odd. Dehaene et al. (1993) were the first to demonstrate that people were consistently faster to respond to relatively smaller magnitudes on the left and larger magnitudes on the right during parity judgment, a phenomenon termed the Spatial Numerical Association of Response Codes—or SNARC—effect. This response pattern is often taken as evidence of a MNL (Dehaene et al., 1993; Fias et al., 1996; Hubbard et al., 2009; but see Nuerk et al., 2015; Proctor and Xiong, 2015; Abrahamse et al., 2016 for recent discussion of alternative explanations). This effect has been demonstrated across many stimulus types (e.g., Nuerk et al., 2005; Ren et al., 2011; Prpic et al., 2018). Furthermore, the SNARC effect is generally viewed as an implicit, quantitative measure of a person’s internal conception of spatially-oriented number and may prove to be useful in illuminating the building blocks of complex mathematical thinking. The distance effect, or the finding that numbers “closer” in numerical magnitude are more difficult to discriminate than those that are “farther” (Moyer and Landauer, 1967; Restle, 1970), is also often taken as evidence of a MNL, though it should be noted that this effect is not sensitive to spatial organization or direction.

The relationship between individual SNARC effects and formal mathematical abilities has become an emerging topic of interest, yet the nature of this relationship is still not well defined. Recent studies of the SNARC have highlighted notable variability in the strength and direction of people’s SNARC effects. Despite group-level effects that indicate a classic SNARC effect, about 20–40% of individuals either have no SNARC effect or one that would suggest a right-to-left SNA (Wood et al., 2006; Cipora and Wood, 2017, Supplementary Material). Unfortunately, attempts to link this variability in SNAs to mathematical proficiency have yielded mostly paradoxical findings, with greater math skill related to weaker or null SNARC effects for whole numbers in adults (Cipora and Nuerk, 2013; Hoffmann et al., 2014) and children (Schneider et al., 2009; Gibson and Maurer, 2016).

However, there has been some evidence that spatial ability may account for these differences. Viarouge et al. (2014) demonstrated that individual differences in the whole number SNARC were explained by measures of spatial cognition and distance effects. Furthermore, a group of professional engineers exhibited significant SNARC effects, while expert mathematicians did not (Cipora et al., 2016; see also Hoffmann et al., 2014). This is further supported by a study of spatial representations of angle magnitude, with engineering students showing SNARC-like effects for angles whereas psychology students did not (Fumarola et al., 2016). This suggests that other factors, such as visuospatial/mental imagery skills or perhaps more domain-general skills rather than domain-specific ones, may be closely linked to the SNARC and act as a mediating factor between MNL representations and math outcomes.

### Number Line Estimation and the Link to Mathematics

While the SNARC effect reveals an implicit link between numerical magnitudes and space, experimental paradigms using physical number lines attempt to more *explicitly* probe participants’ underlying spatial conceptions of number. Perhaps the most common such paradigm is the Number Line Estimation (NLE) task, in which participants place a given number on a physical, horizontally-oriented line that typically includes labeled endpoints (e.g., Siegler and Opfer, 2003). Performance on the task is classically measured in terms of acuity and/or the linear fit of participant responses. This paradigm is widely used in the numerical cognition literature, as it provides a concrete link between physical and mental spatial representations of numerical magnitudes.

Several studies have now demonstrated a link between number line estimation ability and math achievement (Siegler and Opfer, 2003; Booth and Siegler, 2006; Muldoon et al., 2013; Friso-van den Bos et al., 2015; Simms et al., 2016), with greater acuity on NLE tasks associated with higher math ability. These findings have been validated by a recent developmental meta-analysis of such studies (Schneider et al., 2018), which found a strong correlation between number line estimation ability and measures of mathematical competence, including counting, arithmetic, school grades, and standardized test scores. The link between number line estimation and stronger internal magnitude representations has been extended to training studies using linear gameplay elements. Studies of board games that rely heavily on gameplay components reminiscent of number lines, such as *Chutes and Ladders*, have demonstrated a positive effect on a range of mathematically-relevant outcomes (Ramani and Siegler, 2008; Whyte and Bull, 2008; Siegler and Ramani, 2009), including numerical magnitude comparison, counting ability, and more formal number line estimation tasks.

Some scholars contend that the relationship between NLE performance and math proficiency can be attributed to other, related cognitive factors, many of which are spatial in nature. For instance, Simms et al. (2016) found that visuospatial abilities mediated the relationship between linearity of NLE responses and math achievement in children aged 8–10 years. Interestingly, Gunderson et al. (2012) found that number line performance mediated the relationship between spatial skills and early calculation abilities. Taken together, these studies point to the intertwined development of spatial ability and numerical estimation abilities underlying later math achievement.

### The Importance of Fractions

Notably, the entirety of this new research has focused solely on SNAs (and specifically the SNARC effects) for whole numbers. This is surprising, as recent behavioral studies have repeatedly demonstrated links between basic numerical abilities and individual differences in fraction knowledge. In middle school, fraction magnitude knowledge and whole number division have been shown to predict individual differences in both fraction arithmetic and standardized math test scores (Siegler and Pyke, 2013). Furthermore, high-achieving students are more likely to rely on overall (holistic) fraction magnitude when doing fraction tasks, while low achievers are more likely to focus on the components, supporting the hypothesis that stronger holistic mental representations of fraction magnitudes leads to higher levels of overall math achievement (for similar evidence related to math learning disabilities, see Mazzocco et al., 2013). DeWolf et al. (2015) demonstrated that measures of *relational* fraction knowledge and placing decimals onto number lines were the best predictors of algebra performance. The predictiveness of relational fraction concepts may be supported by an underlying ratio-processing system (RPS), which is sensitive to non-symbolic ratios such as line length comparisons (Lewis et al., 2015). Acuity of the RPS is also related to formal math achievement, including performance on symbolic fraction tasks and algebra achievement scores (Matthews et al., 2016), bolstering the claim that holistic fraction magnitude processing is key for later math learning.

As evidence emerges that fractions provide a foundation for later achievement in mathematics, researchers have also begun to investigate the developmental predictors of elementary school children’s fraction knowledge. A longitudinal study by Ye et al. (2016) demonstrated the importance of number line estimation, division and multiplication with whole numbers, as well as non-symbolic proportional reasoning, on later fraction knowledge. Additionally, Schneider et al. (2018) found that the relationship between NLE and math achievement became stronger with age, a pattern that could be attributed to fraction knowledge. Jordan et al. (2013) found that performance on a number line estimation task was the *largest* independent contributor to both conceptual and procedural fraction knowledge, highlighting the importance of SNAs for fraction understanding. As a number line estimation task is essentially an explicit measure of internal representations of the number line, this finding indicates that an implicit measure of SNAs (e.g., the fraction SNARC) might be similarly sensitive.

In line with this prediction and previous work on the SNARC effect for whole numbers, fractions have indeed elicited a group-level classic SNARC effect (Toomarian and Hubbard, 2018b). Inasmuch as whole number SNAs may be related to spatial or math-related outcomes, inter-individual variability in the fractions SNARC may be an important signature of differences in holistic fraction processing and mathematics ability more broadly. However, the link between the fraction SNARC and individual differences in math achievement has not yet been explored. Furthermore, no studies have investigated the possibility that a more explicit number line estimation task may mediate the relationship between the implicit fractions SNARC effect and spatial/mathematical measures. While Schneider et al. (2009) found that a parity based SNARC effect for whole numbers did not predict conceptual knowledge of decimal fractions and that a decimal NLE task did, it is unclear whether these findings would hold if fractions were used to elicit a SNARC instead. An independent effect of the fractions SNARC on mathematical outcome measures would further support the critical role of spatial processing in fraction processing and proportional reasoning (Möhring et al., 2015).

### The Present Study

This study aimed to investigate the link between implicit spatial representations of fractions in adults and explicit measures of numerical/mathematical knowledge by focusing on three central questions: (1) which factors predict individual differences in spatial representations of fractions? (2) to what extent is the SNARC effect distinct from other indices of numerical processing (e.g., the distance effect and number line estimation) and (3) do spatial representations of fractions, as measured by the fractions SNARC and NLE task, uniquely account for differences in math achievement in university undergraduates?

With respect to the first two research questions, our predictions were largely influenced by theoretical considerations. If people consistently rely on the MNL when comparing numerical magnitudes, that would imply (1) that SNARC effects are distinct from other basic factors, such as IQ, and (2) associations between the distance effect, SNARC effect, and performance on a number line estimation task. As for whether the fractions SNARC and NLE performance would predict math achievement in our sample, we did not have strong *a priori* predictions due to the conflicting nature of relevant theory and past research. Theoretically, a stronger internal spatial-numerical representation (i.e., MNL) should be associated with higher mathematical achievement. Additionally, non-symbolic ratio comparison has been shown to predict university algebra scores (Matthews et al., 2016), and NLE performance has been associated with greater mathematical competence (Schneider et al., 2018). However, the SNARC effect with whole numbers has not been positively associated with math proficiency (e.g., Hoffmann et al., 2014; Cipora et al., 2016). In light of these inconsistent findings, we hypothesized that the slope of participants’ fraction SNARC effects and NLE performance might uniquely account for variability in more domain-specific outcome measures, such as a formal test of fraction knowledge and a standardized measure of basic math skills, but would not predict algebra scores.

## Methods and Measures

### Participants and Procedure

One hundred and six undergraduate students were recruited for this study. However, no data was collected for one participant, as the session was disrupted shortly after the start. Thus, the final sample consisted of 105 adults, aged 18–43 (mean = 20.39 years, *SD* = 2.83), who participated in this study for course credit. All components of the study were approved by the Institutional Review Board (IRB#2013-1346). Computerized experiments were programmed with E-prime 2.0.8.90a (Psychology Software Tools, Sharpsburg, PA, United States) on a Dell Optiplex 390 Desktop PC (3.1 GHz, 4 GB RAM) running Windows 7.0 64-bit operating system. Visual stimuli were presented on a Dell UltraSharp U2212H 21.5″ flat-screen monitor at a resolution of 1024 × 768 and a refresh rate of 60 Hz.

### Measures

The study session lasted approximately 1.5 h, during which time participants completed several measures, in following order:

#### Fraction Comparison

Participants compared all 26 single-digit, irreducible fractions to the standard fraction ½, indicating with a keyboard response if the fraction was larger or smaller than the standard. In an exact replication of Experiment 2 from Toomarian and Hubbard (2018b), each fraction appeared eight times, with response side counterbalanced across two blocks and two different run orders. A total of 10 practice trials preceded each block, which included visual feedback. A central fixation cross appeared for 600 ms, followed by a blank screen for 1000 ms and the target fraction for 3000 ms or until a response was detected. Fraction stimuli were approximately 1.8 cm wide and 2.7 cm tall (1.5° × 2.8° visual angle). Left button presses corresponded to the ‘d’ key, and right button presses corresponded to the ‘k’ key on the QWERTY keyboard (distance = 8.5 cm).

Left hand median reaction times were subtracted from left hand median reaction times for each fraction magnitude for each participant. These differences in reaction times (dRT) were regressed on fraction magnitude, resulting in either a positive or negative sloping regression line for each participant (Lorch and Myers, 1990; Fias et al., 1996). Negative slopes indicate a classic SNARC effect (small magnitudes associated with the left, large with right), and positive slopes indicate the reverse. Data from this task yielded several outcome measures: an individual SNARC effect, individual distance effect, overall RT, and overall accuracy. It is important to note that this task is based on a direct magnitude comparison rather than the classic parity judgment primarily because fractions cannot be classified as even or odd.

#### Number Line Estimation (NLE)

This computerized number-to-position task included both proper fractions on a 0–1 number line and improper fractions on a 0–5 number line (adapted from Torbeyns et al., 2015). Specifically, participants estimated the position on a number line that corresponded with the fraction displayed at the top of the screen. On the basis of these estimates, we calculated the percent absolute error (PAE) score for each participant (PAE = [| answer – correct answer| /numerical range]). Thus, smaller PAE values indicate higher acuity for fractions.

#### Fraction Knowledge Assessment (FKA)

This written assessment of fraction knowledge is comprised of items largely drawn from the TIMSS and NAEP (Matthews et al., 2016). Items were intended to assess both procedural (e.g., “^1^/_10_ + ⅗ = __”) and conceptual (e.g., “How many fractions are possible fractions are between ¼ and ½?”) fraction knowledge. The assessment had a total possible score of 38 points; percentage correct was used as a quantitative measure of general fraction knowledge for each participant.

#### Wechsler Abbreviated Scale of Intelligence, Second Edition (WASI-II)

This standardized assessment was used to quickly generate an estimate of IQ. Administration of two subtests—Vocabulary and Matrix Reasoning (MR)—yielded the Full Scale IQ 2 (FSIQ-2). Scores for Matrix Reasoning were also used as a measure of abstract problem solving, inductive reasoning and spatial reasoning.

#### Placement Exams

Participants provided consent for the study team to obtain placement test scores from university administration. All students entering the University of Wisconsin system take a required series of math and English placement tests, comprised of Basic Mathematics, Algebra, Trigonometry, English, and Reading scores. Of particular theoretical interest are the Basic Math and Algebra scores, which have strong internal consistency (Cronbach’s α = 0.90) and have been linked to non-symbolic ratio processing ability (Matthews et al., 2016). Scores are standardized on a scale ranging from 150 to 850 points.

## Results

The accuracy threshold for inclusion was 80%, but all participants who completed the session exceeded this threshold. Missing data due to various technical issues (e.g., computer error, fire alarms) resulted in several participants without data for all of the measures conducted in a session. Additionally, placement test scores were unavailable for 19 participants. Thus, the following analyses describe results from slightly different samples, dependent on which measures were available for each participant. Sample sizes for each analysis are listed in Table 1, along with descriptive statistics. Diagnostic analyses revealed two influential points (as measured by Cook’s *d*). These outlier points reflected extreme but not implausible values, and removal of these two points did not meaningfully change the regression results. Thus, all possible data points were retained in the following models. SNARC effects were analyzed using regression analyses of repeated-measures data and *t*-tests against zero. This method has come to be favored over using an ANOVA as magnitudes can be analyzed continuously and accounts for between-subjects variability (for additional rationale on this approach, see Fias et al., 1996). This approach is particularly useful for investigations of individual differences, as it yields a SNARC slope for each participant which can then be used in further analyses (e.g., correlations). Due to incongruous scaling of the measures, all reported beta values reflect standardized regression coefficients. Outcome measures were not standardized. There was no evidence of multicollinearity among the factors included in the model, as evidenced by variance inflation factors less than 10.

**Table 1:** Descriptive statistics.

Measure	*n*	Mean (*SD*)
Fraction comparison		
Reaction time (RT)	99	749.44 (137.24)
Accuracy (ACC)	99	0.96 (0.02)
SNARC slope (SNARC)	99	–75.57 (276.32)
Distance Effect slope (DIST)	99	–912.85 (373.67)
Fraction Knowledge Assessment % (FKA)	100	84.11 (10.28)
Number Line Estimation (PAE)	94	6.89 (2.75)
Algebra Exam (ALG)	86	585.00 (101.80)
Basic Math Exam (MBSC)	86	629.19 (104.87)
WASI- Full-Scale IQ (FSIQ)	102	104.33 (10.50)
Matrix Reasoning (MR)	102	49.81 (8.26)
Vocabulary (VOCAB)	102	55.36 (6.57)


*Descriptions of each measure include the abbreviation used in subsequent analyses. Reaction time measured in milliseconds. SNARC, spatial-numerical association of response codes.*

### Distance and SNARC Effects

As predicted, there was a significant group-level distance effect, both when average RTs were regressed on magnitude (β = -840.11, *F*[1,11] = 105.8, *p* < 0.001) and when individual distance effects were tested against zero in a one-sample *t*-test (β = -912.85, *t*[1,98] = -24.31, *p* = 0.007). Consistent with Toomarian and Hubbard (2018b), individual SNARC slopes were overall significantly less than zero (β = -75.57, *t*[1,98] = -2.72, *p* < 0.001), indicating a group-level classic SNARC effect for fractions.

### Correlational Analyses

Simple bivariate correlations for all measures in the study are listed in Table 2. There was no correlation between the distance effect and SNARC effect (*r* = 0.05, *p* = 0.622). When accounting for the possible mediating role of RT, the correlation was still non-significant (*p* = 0.54). The fractions SNARC was correlated with both acuity on the NLE task (PAE; *r* = 0.23, *p* = 0.029) and basic math ability (MBSC, *r* = -0.26, *p* = 0.018), meaning that increasingly negative SNARC slopes were associated with lower PAE scores (greater acuity) on the fractions NLE task and better basic math scores. Lower PAE was also associated with higher scores on the fractions task (FKA; *r* = -0.42, *p* < 0.001), higher accuracy on the fraction comparison task (ACC; *r* = -0.33, *p* = 0.001), basic math scores (*r* = -0.26, *p* = 0.024), and algebra scores (ALG; *r* = -0.26, *p* = 0.023).

**Table 2:** Bivariate correlations.

	FKA	SNARC	FSIQ	RT	ACC	DIST	PAE	MBSC	ALG	MR
SNARC	–0.15	1								
FSIQ	0.26**	–0.09	1							
RT	–0.14	–0.01	0.06	1						
ACC	0.26**	0.01	0.11	0.20*	1					
DIST	0.09	0.05	0.03	–0.69***	–0.27**	1				
PAE	–0.42***	0.23*	0.02	0.19	–0.33**	–0.06	1			
MBSC	0.43***	–0.26*	0.36***	–0.02	0.09	–0.06	–0.26*	1		
ALG	0.33**	–0.17	0.33**	–0.18	0.15	0.09	–0.26*	0.70***	1	
MR	0.26**	–0.13	0.86***	–0.01	0.10	0.12	–0.07	0.29**	0.34**	1
Vocab	0.18	–0.01	0.76***	0.11	0.07	–0.04	0.10	0.33**	0.18	0.36***


*^∗∗∗^*p* < 0.001, ^∗∗^*p* < 0.01, ^∗^*p* < 0.05.*

### Predicting the SNARC Effect

To investigate our first research question of which factors predict the SNARC effect, we used linear regression to model the following equation: SNARC_i_ = α + β_1_ MR + β_2_ Vocab + β_3_ PAE + β_4_ RT + β_5_ ACC + ε (see Table 3). The only significant factor in the specified model was performance on the number line estimation task. When holding all other factors constant, for every standard deviation increase in PAE (i.e., decreasing acuity), the SNARC slope is expected to increase by 82.88 (*t* = 2.76, *p* = 0.007), resulting in an increasingly positive slope. In other words, acuity for a physical number line task—as measured by PAE—uniquely predicts the degree to which participants activate holistic fraction magnitudes on their (implicit) mental number line. Indices of general intelligence, RTs, and accuracy did not meaningfully influence the fraction SNARC. This provides some validation that the fraction SNARC effect is a valuable measurement of internal SNAs and is distinct from other measures of task performance. However, this model predicted relatively little variance in SNARC slopes, suggesting that other factors (not measured in this investigation) have greater influence on the variability in individuals’ SNARC effects.

**Table 3:** Regression analysis for variables predicting SNARC effect slope.

Variable	β	*SE*
Intercept	–74.51	28.31
WASI- MR	–15.71	32.42
WASI - Vocab	–8.85	30.43
Number Line Est. (PAE)	81.12*	31.10
RT	–10.95	29.95
ACC	52.00	30.77
R-squared	0.086	
Adjusted R-Squared	0.032	


*^∗^p < 0.05.* β *represents standardized regression coefficients. *n* = 90.*

### Contributions to Fraction Knowledge

Next, we aimed to test the unique contributions of SNARC slopes and PAE to procedural and conceptual fraction knowledge, as measured by the FKA. To do this, we conducted a three-step hierarchical regression analysis that introduced SNARC and then PAE to the reduced model containing other basic cognitive factors that could influence FKA scores (see Table 4). Because participants with any missing values for SNARC, PAE or FKA were excluded from analysis, 88 participants were retained for this analysis. Step 1 included only mean RT, mean accuracy, and full scale IQ, which together accounted for 14% of the variance in FKA scores (*F*[3,84] = 5.45, *p* = 0.002). All of these factors on their own predicted FKA scores. When SNARC slopes were added in Step 2, only an additional 1% of variance in FKA scores was accounted for, and it was not significantly improved from the reduced model (*F*[1,83] = 3.003, *p* = 0.09). In the third step, PAE from the NLE task was added to the model, which increased the amount of explained variance in FKA scores to 23%, a significant improvement in model specification (*F*[1,82] = 9.35, *p* = 0.003) compared to the model in Step 2.

**Table 4:** Hierarchical regression analysis for variables predicting FKA score.

	Regression 1		Regression 2		Regression 3	
Predictor variable	β	*SE*	β	*SE*	β	*SE*
RT	–0.02	0.01	–0.02*	0.01	–0.01	0.01
ACC	0.03**	0.01	0.03**	0.01	0.02	0.01
FSIQ	0.02*	0.01	0.02*	0.01	0.03*	0.01
SNARC			–0.02	0.01	–0.01	0.01
Number Line Est. (PAE)					–0.03**	0.01

*R*^2^		0.13		0.15		0.23
Δ*R*^2^				0.02		0.07**


*^∗^*p* < 0.05, ^∗∗^*p* < 0.01, all reported *R*^*2*^ are adjusted. *n* = 88.*

Notably, there was no evidence of multicollinearity among the factors included in the model, as evidenced by relatively small variance inflation factors (SNARC slope = 1.16; PAE = 1.29; RT = 1.17, ACC = 1.35, IQ = 1.03). When all other basic cognitive factors and the SNARC are controlled for, FKA scores decrease by 0.03 points for each standard deviation increase in PAE for the fractions number line task. To summarize, scores on a fraction test were significantly predicted by an explicit number line estimation task but not by an implicit measure of SNAs for fractions, contrary to our initial hypothesis.

### Contributions to Basic Math Skills

To investigate the relative contributions of implicit and explicit processing of SNAs to basic math skills, we conducted another three-step hierarchical regression analysis, with progressive introduction of the SNARC effect and then PAE score as predictors. The first model contained the same initial predictors as the previous model for FKA scores, namely RT, ACC, and FSIQ (see Table 5). Because participants with any missing values for SNARC, PAE or MBSC were excluded from analysis, 73 participants were retained for this analysis.

**Table 5:** Hierarchical regression analysis for variables predicting basic math score.

	Regression 1		Regression 2		Regression 3	
Predictor Variable	β	*SE*	β	*SE*	β	*SE*
RT	–5.10	11.87	–7.29	11.95	–1.98	11.97
ACC	2.01	11.74	4.96	11.92	–4.79	12.61
FSIQ	38.19**	13.62	36.35**	13.64	38.31**	13.37
SNARC			–15.52	12.35	–8.72	12.53
Number Line Est. (PAE)					–26.69*	13.14

*R*^2^		0.07		0.08		0.11
Δ*R*^2^				0.01		0.03*


*^∗^*p* < 0.05, ^∗∗^*p* < 0.01, all reported *R*^*2*^ are adjusted. *n* = 73.*

This first regression model explained 7% of the variance in scores for basic math skills (*F*[3,69] = 2.78, *p* = 0.05). In this reduced sample, only FSIQ predicted scores on MBSC, meaning that when holding all other factors constant, each standard deviation increase in FSIQ is associated with a 38.19 point increase in MBSC score. The addition of SNARC slopes explained 1% more variance, though according to a partial F-test, this model was not a significant improvement (*F*[1,68] = 1.58, *p* = 0.21). The last step—adding in PAE—resulted in a slightly better model and explained an additional 3% of variance in MBSC scores (*F*[1,67] = 4.13, *p* = 0.05). For each standard deviation increase in PAE (indicating reduced acuity), MBSC scores decrease by 26.69 points, controlling for changes in ACC, RT, FSIQ, and SNARC.

### Contributions to Algebraic Knowledge

The last outcome measure we tested was score on a standardized algebra exam. This outcome measure was motivated by findings that college students’ non-symbolic ratio judgments significantly predicted algebra placement exam scores (Matthews et al., 2016). To test whether either the SNARC or PAE predicted algebra scores, we conducted another three-step hierarchical regression analysis to investigate the relative contributions of implicit and explicit measures of SNAs to ALG. These models followed the same structure as the previous two hierarchical regression models, with basic cognitive factors in the initial model, followed by progressive introduction SNARC and PAE score (Table 6). Due to incomplete cases, 73 participants were retained for analysis.

**Table 6:** Hierarchical regression analysis for variables predicting algebra scores.

	Regression 1		Regression 2		Regression 3	
Predictor Variable	β	*SE*	β	*SE*	β	*SE*
RT	–28.35	10.49**	–29.19	10.66**	–25.57	10.82*
ACC	17.02	10.38	18.15	10.64	11.50	11.39
FSIQ	23.59	12.04	22.88	12.17	24.22	12.08*
SNARC			–5.95	11.02	–1.32	11.32
Number Line Est. (PAE)					–18.19	11.87

*R*^2^		0.12		0.11		0.13
Δ*R*^2^				–0.01		0.02


*^∗^*p* < 0.05, ^∗∗^*p* < 0.01, all reported *R*^*2*^ are adjusted. *n* = 73.*

In the initial model, only RT was a significant predictor of algebra test scores (*p* = 0.008), and 12% of the variance in ALG was explained by the model. When SNARC was introduced, the model actually explained less variance, when the number of factors was considered (adj-*R*^2^ = 0.11). Adding PAE to the model explained an additional 1% of variance from the first model, though neither of the subsequent models were any better than the first (*1* vs. *2*: *F*[1,68] = 0.29, *p* = 0.59; *2* vs. *3*: *F*[1,67] = 2.35, *p* = 0.13), indicating that neither implicit not explicit measures of SNAs have predictive power over algebra test scores. In the final model, only RT and FSIQ significantly predicted ALG. Thus, while holding all other variables in the final regression constant, ALG scores increase by 25.57 points for every standard deviation decrease in RT; they increase by 24.22 points for every standard deviation increase in FSIQ.

### Mediation Analyses

Despite the extensive planned analyses, it is unclear whether SNARC slopes and PAE scores contribute uniquely to our outcomes of interest, specifically FKA and MBSC scores. We employed mediated path analyses to determine whether acuity on the NLE task—as measured by PAE—mediated the relationship between the SNARC and our two outcome measures of interest. We did not have reason to believe that there was any mediation in the case of ALG scores, since neither measure was predictive of ALG scores in prior analyses. Additionally, while the independent variable predicting the dependent variable is often regarded as a necessary condition for conducting mediation analyses (Baron and Kenny, 1986), recent guidelines have supported mediation analysis without such a relationship in certain cases (Shrout and Bolger, 2002). For instance, in cases when theory would predict such a relationship and sample sizes are relatively small, mediation analysis may be conducted with bootstrapped confidence intervals. Thus, although SNARC did not predict FKA scores, we proceeded with mediated path analysis nonetheless. To test whether PAE mediates the relationship between SNARC and our two dependent measures (FKA and MBSC), we conducted path analysis with mediation using the ‘lavaan’ package in R (Rosseel, 2012). Variables are unstandardized. We used the full information maximum-likelihood imputation approach for missing values.

**FIGURE 1:**
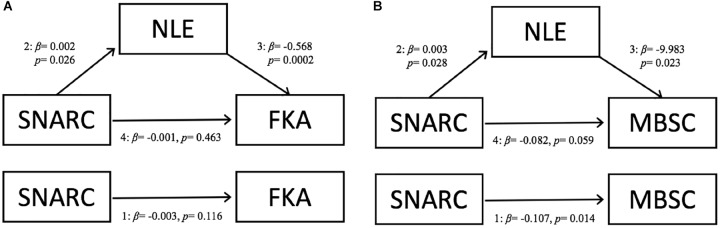
Schematics of the path analyses testing whether number line estimation performance mediates the relationship between SNARC and **(A)** fraction knowledge, or **(B)** basic math ability. SNARC, Spatial-Numerical Association of Response Codes; FKA, Fraction Knowledge Assessment; NLE, Number Line Estimation, representing percent absolute error (PAE) values; MBSC, Basic Math.

In Model A (Figure 1), the only direct effect was between NLE and FKA scores; adjusting for SNARC slopes, every 1-unit increase in PAE is associated with a decrease of *b* = 0.568 (*SE* = 0.16, *p* < 0.001) in FKA score. There was no indirect effect, and thus no evidence of full mediation *ab* = -0.001 (*SE* = 0.0008, *p* = 0.204). A bias-corrected bootstrapped 95% confidence interval based on 10,000 samples included zero [-0.003, 0.0001], confirming that there is no evidence of mediation in this model.

In Model B, we tested for mediation between SNARC and MBSC score. Independent of PAE, a one-unit increase in SNARC slope is associated with 0.107 decrease in MBSC score (*SE* = 0.044, *p* = 0.014). Every unit increase in SNARC slope is associated with an *a* = 0.003 (*SE* = 0.001, *p* = 0.028) increase in PAE on the NLE task. Adjusting for SNARC slopes, every 1-unit increase in PAE is associated with a decrease of *b* = 9.983 (*SE* = 4.400, *p* = 0.023) in MBSC score. There was no indirect effect, and thus no evidence that PAE score mediated this association *ab* = -0.026 (*SE* = 0.019, *p* = 0.184). A bias-corrected bootstrapped 95% confidence interval based on 10,000 samples included zero [-0.077, 0.0002], confirming that there is no evidence of full mediation in this model. However, there was a significant total effect for the model (*SE* = 0.044, *p* = 0.015), indicating that the model fit the data well and is evidence that PAE may at least partially mediate the relationship between SNARC and MBSC.

## Discussion

In this study, we investigated the relationship between implicit and explicit measures of SNAs, including the link to formal math abilities. First, we successfully replicated our previous work demonstrating that a classic SNARC for fraction magnitudes emerges at the group-level (Toomarian and Hubbard, 2018b) and for the majority of adult individuals. This replication in a separate, larger sample of adults supports the assertion that people can and do represent fractions holistically under appropriate task constraints.

We then moved past group level effects to investigate a second question: which factors influence individual differences in participants’ SNARC effects. Performance on a number line estimation task, which included whole numbers and fractions, was uniquely predictive of individual SNARC slopes. Importantly, this relationship emerged even while controlling for factors such as response time, overall accuracy, and two IQ subtests. That accuracy and RT in the comparison task were not associated with SNARC slopes indicates that the SNARC is measuring a unique, spatial ability that cannot be accounted for by basic processing speed or ability to do the task. These results are theoretically supported by the MNL hypothesis; if the SNARC is a measure of reliance on a right-to-left spatially oriented MNL, greater reliance on this internal number line (evidenced by more negative SNARC slopes) should be related to acuity on a similarly oriented, external number line task. However, Schneider et al. (2009) found no relationship between NLE performance and the parity SNARC in kids, thereby challenging this interpretation of the results. Instead, they argue that the internal and external number line cannot be equated, at least early in development.

Our results indicate that NLE has greater predictive power than the SNARC for multiple outcome measures, which suggests some degree of dissociation between these two measures. One explanation for this dissociation may be that the fractions SNARC, by nature of being more implicit than the NLE task, has a weaker effect and may not have much influence to exert on explicit outcome measures. This is in contrast to the NLE task, which has both theoretical (e.g., Siegler et al., 2011) and empirical (e.g., Thompson and Siegler, 2010; Gunderson et al., 2012; Resnick et al., 2016; Ye et al., 2016) support for its role in fractions learning and math proficiency. A recent study demonstrated that number line training but not area model training improved performance on an untrained fraction magnitude comparison task, highlighting the utility of an external spatial-numerical representation (Hamdan and Gunderson, 2017).

In this study, there was no evidence of a correlation between the distance effect and SNARC effect. Previous studies with whole numbers have yielded mixed evidence on the relationship between the distance and SNARC effects; Viarouge et al. (2014) found a correlation between these measures, while Gibson and Maurer (2016) did not. Interestingly, Schneider et al. (2009) found a significant correlation in one experiment, but not in a subsequent experiment.^1^ While both effects are often taken as evidence supporting the MNL hypothesis, there is a key difference between the two effects: only the SNARC effect reflects a directional/spatialized association. With this difference in mind, it is not difficult to imagine that these effects might dissociate within subjects, particularly for stimuli such as common fractions, for which the cognitive processing mechanisms are still not well understood.

Lastly, neither the fractions SNARC nor PAE predicted algebra placement exam scores, despite PAE being a significant predictor of fraction knowledge and basic math skills. This suggests that more implicit processing of spatial-numerical representation may not be as readily recruited during higher-order mathematical concepts, but rather may serve as a foundation for thinking about simpler problems involving rational magnitudes. This would cohere well with the recent finding that the ability to place decimals, but not fractions, on number lines was one of the best predictors of algebra performance (DeWolf et al., 2015).

### Limitations

Here we would like to note several aspects of the current research that may limit the interpretability of the results. First, as previously mentioned, the sample size was moderately reduced for each analysis due to missing data points across various measures. This issue was perhaps most significant for the hierarchical regressions with MBSC and ALG as the dependent variables, since the placement tests were the variables for which there were the most missing data points. While this reduction affected the degrees of freedom, decreased the adjusted R-squared, and increased the possible influence of outliers, it is important to note that the total *n* never dipped below the number required for a medium effect size and there were no marginal effects.

Additionally, recent simulation work on detecting reliable SNARC effects with various sample sizes, stimulus repetitions, and effects has provided guidelines for obtaining results of moderate effect (Cipora and Wood, 2017). Specifically, studies are recommended to test a minimum of 20 participants and with twenty repetitions per stimulus. While our sample size exceeds this minimum requirement, there are only eight repetitions per stimulus in the task from which we draw our individual SNARC slopes. That said, our stimulus set contains four times the number of individual numerical stimuli as classic SNARC paradigms (24 vs. 8), thus offsetting the reduction in the number of trials per stimulus. Thus, the overall experiment time would be unreasonably long if we were to collect twenty observations per stimulus per condition and would thus compromise the integrity of the data. Furthermore, because this recommendation stems from the desire to control for intra-individual variability, we argue that our wide range of fraction magnitudes in fact serves a similar purpose; by increasing the number of points on the MNL to which participants are asked to respond, we are effectively controlling for this variability in an analogous fashion.

## Conclusion

In this study, we investigated how individual spatial representations of fractions relate to explicit fraction knowledge and two other formal measures of math achievement. We observed significant group-level SNARC and distance effects based on overall fraction magnitude, with notable individual variability. Performance for the number line estimation task was correlated with SNARC slopes and predicted significant variance in SNARC slopes even when accounting for factors such as overall accuracy and matrix reasoning ability. Multi-step regressions revealed that NLE performance was a significant predictor of fraction test scores and basic math skills but the SNARC was not, indicating that working with an explicit number line may be a stronger predictor of domain-specific and domain-general math abilities than more implicit number line processing of fractions. Neither individual SNARC effects nor NLE performance were significant predictors of algebra scores. This suggests that the MNL may not be as readily recruited during higher-order mathematical concepts, but rather may be a foundation for thinking about simpler problems involving rational magnitudes.

The current study informs our understanding of the relative contributions of more implicit (SNARC) and explicit (NLE) processing of fractions, but it is still unknown whether these relations are consistent from childhood to adulthood. Developmental studies—particularly with continuous age data—are necessary to better understand how spatial and numerical conceptions influence mathematical thinking. Future studies should investigate this relationship with (1) a larger, more educationally-diverse sample, and (2) additional spatial tasks as covariates.

## Ethics Statement

This study was carried out in accordance with the recommendations of the University of Wisconsin-Madison Institutional Review Board (IRB#2013-1346) with written informed consent from all subjects. All subjects gave written informed consent in accordance with the Declaration of Helsinki. The protocol was approved by the Educational/Social Behavioral Sciences (Ed/SBS) IRB at UW-Madison.

## Author Contributions

ET and EH conceptualized and designed the study. ET collected the data. ET and RM analyzed the data. ET wrote the first draft of the manuscript. All authors revised, read, and approved the submitted version of manuscript.

## Conflict of Interest Statement

The authors declare that the research was conducted in the absence of any commercial or financial relationships that could be construed as a potential conflict of interest.

## Footnotes

1 Beyond just significance testing, these studies also found markedly different correlation coefficients for the relationship between SNARC and distance effect: Viarouge et al. (2014): *r* = 0.52; Schneider et al. (2009): *r* = 0.25 (Experiment 1) and *r* = -0.03 (Experiment 2); Gibson and Maurer (2016): *r* = -0.06; the current study: *r* = 0.05.
